# An Open Trial of Metacognitive Remediation Therapy and Pharmacotherapy to Promote Smoking Cessation among Individuals with Psychotic-Spectrum Disorders

**DOI:** 10.1155/2021/6617716

**Published:** 2021-07-27

**Authors:** Nicholas J. K. Breitborde, Brittney Keller-Hamilton, Aubrey M. Moe, Jacob G. Pine, Nicholas Nelson, David Weiss, Tory Hogan, Amanda Quisenberry, Andreas Teferra, Amy K. Ferketich

**Affiliations:** ^1^The Ohio State University, USA; ^2^Southeast, Inc., USA; ^3^Roswell Park Comprehensive Cancer Center, USA

## Abstract

**Introduction:**

Individuals with psychotic-spectrum disorders may smoke due to the ameliorating effect of nicotine on the cognitive deficits that accompany these illnesses. Metacognitive remediation therapy (MCR) has been shown to produce improvements in cognitive functioning among individuals with psychotic-spectrum disorders and provides a foundation for a novel smoking cessation intervention for this population.

**Aims:**

To complete an open investigation of pharmacotherapy and a modified version of MCR [MCR to Quit (MCR-Q)] in promoting smoking cessation among individuals with psychotic-spectrum disorders.

**Methods:**

Forty-nine individuals with a psychotic-spectrum disorder and who currently smoke cigarettes participated in MCR-Q while also receiving evidence-based smoking cessation pharmacotherapy. Tobacco use was assessed as follows: (i) prior to MCR-Q, (ii) immediately after completing MCR-Q, and (iii) six weeks after completion of MCR-Q.

**Results:**

/*Findings*. During participation in MCR-Q, nearly 80% of participants made a 24-hour quit attempt. Following the completion of MCR-Q, participants experienced reductions in level of nicotine dependency and exhaled carbon monoxide, with reductions in nicotine dependency sustained six weeks after completion of MCR-Q. Over the course of their participation in MCR-Q, participants reported strong therapeutic alliance with their MCR-Q therapist and high levels of intrinsic motivation with regard to completing MCR-Q exercises.

**Conclusions:**

The results from the current study suggest cautious optimism with regard to the use of MCR-Q in combination with medication for individuals with psychotic-spectrum disorders who want to quit smoking.

## 1. Introduction

Individuals with psychotic-spectrum disorders, such as schizophrenia or mood disorder with psychosis, have a mortality rate that is over three times greater than the general population [1]. Tobacco use contributes to this disparity, with recent data suggesting that 64-79% of individuals with a psychotic-spectrum disorder regularly smoke tobacco [2] and that over 50% of the deaths among individuals with psychotic-spectrum disorders can be attributed to tobacco use [1, 3]. Contributing to this health disparity is limited access to smoking cessation treatments—only 4% of individuals with serious mental illnesses (e.g., psychotic-spectrum disorders) who smoke receive smoking cessation assistance from a health provider [4].

Clinical guidelines for smoking cessation programs recommend a combination of medication and counseling [5]. Although cessation pharmacotherapy can be effective in helping smokers with mental illness quit, a meta-analysis demonstrated that existing counseling interventions are not effective [6]. As individuals with psychotic-spectrum disorders may smoke due to the ameliorating effect of nicotine on the cognitive deficits that accompany these illnesses [7], the ineffectiveness of cessation counseling interventions within this population may stem in part from their inability to address cognitive functioning [8]. In particular, a recent meta-analysis demonstrated that individuals with schizophrenia who smoke experience greater impairments in attention, learning, problem-solving, processing speed, and working memory as compared to individuals with schizophrenia who do not smoke [9] suggesting that these cognitive domains may represent putative targets for smoking cessation interventions designed to address cognitive functioning among individuals with psychotic-spectrum disorders.

Outside of the smoking cessation literature, metacognitive remediation therapy (MCR: [10]) has shown promise as a novel form of individual psychotherapy for individuals with psychotic-spectrum disorders. MCR is among the growing number of individual psychotherapies whose primary therapeutic aim is improving metacognitive abilities (e.g., [11, 12]) and applies existing strategies for improving metacognition included in psychotherapeutic and educational interventions [13–17] to address two key components of metacognitive abilities: (i) knowledge of cognition (i.e., the ability to identify problem-solving solutions, know how to apply these solutions, and be able to determine which possible solution would be best to use to solve a given problem) and (ii) regulation of cognition (i.e., the ability to plan the use of problem-solving strategies, monitor the success of these strategies during their implementation, and accurately evaluate the success of these strategies after their implementation). Among individuals with first-episode psychosis, MCR has been shown to produce improvements in metacognitive functioning [10] with additional downstream benefits on cognitive, social, and educational/occupational functioning that exceeded those produced by “drill and practice” computerized cognitive remediation [18–20]. These benefits include improvements in each of the five domains of cognitive functioning in which individuals with psychotic-spectrum disorders who smoke experience greater impairments than individuals with psychotic-spectrum disorders who do not smoke (i.e., attention, learning, problem-solving, processing speed, and working memory; [9]).

Given MCR's ability to enhance cognition among individuals with psychotic-spectrum disorders, this study's goal was to complete an open investigation of pharmacotherapy and a modified version of MCR [MCR to Quit (MCR-Q)] to promote smoking cessation among a sample of individuals with psychotic-spectrum disorders (i.e., schizophrenia-spectrum disorders and mood disorders with psychotic features [21]). Given the exploratory nature of this study, analyses focused on smoking-related outcomes, participants' perception of MCR-Q, treatment fidelity, and symptomatology.

## 2. Methods

Written consent was obtained from all participants only after they were provided with a full description of study procedures. The study was conducted in accordance with the Declaration of Helsinki and was approved by the Ohio Department of Health Institutional Review Board.

### 2.1. Participants

**Figure 1:**
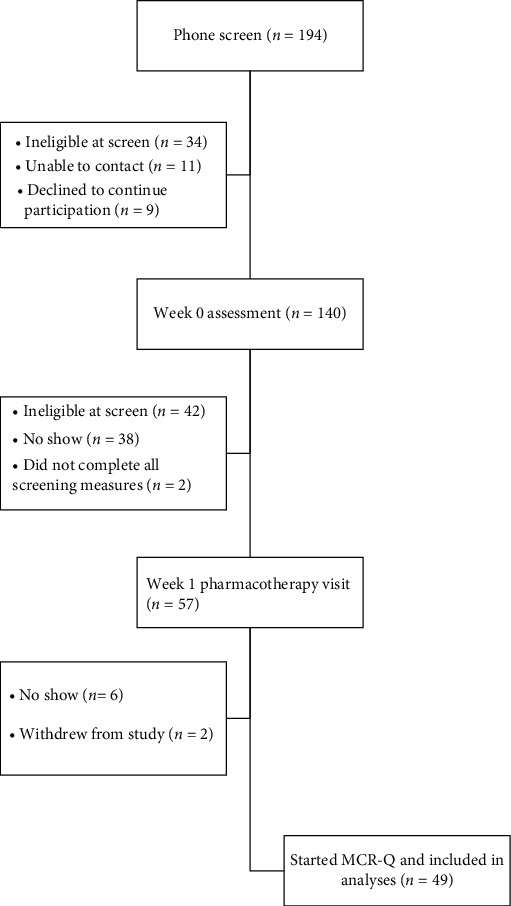
Participant flowchart.

Between October 2017 and August 2018, forty-nine individuals with a psychotic-spectrum disorder who were receiving mental health care at a local community mental health center participated in the study—see Figure 1 for participant flowchart. Eligibility criteria included (i) diagnosis of a schizophrenia-spectrum disorder or bipolar disorder with psychotic features as determined using the Structured Clinical Interview for the DSM-5 [22], (ii) age 18-64 years, (iii) on a stable dose of antipsychotic medication for ≥2 weeks, (iv) fluent in English, (v) current smoker of ≥5 cigarettes per day, (vi) able to provide informed consent, (vii) actively engaged in mental health care defined as having received care continuously for ≥4 months at the community mental health center in which the study took place, and (viii) interested in quitting smoking in the next 30 days. Individuals were excluded from study participation if they (i) met DSM-5 criteria for an active substance use disorder not in remission, (ii) had a premorbid IQ ≤ 70 as estimated using the reading subtest of the Wide Range Achievement Test [23], or (iii) had previously participated in a cognition-enhancing intervention such as MCR.

### 2.2. Interventions

#### 2.2.1. Metacognitive Remediation Therapy to Quit (MCR-Q)

As originally designed, MCR is a six-month intervention that is among the growing number of psychotherapies whose therapeutic target is improvements in metacognitive abilities [24]. Participants complete two sessions per week over the six-month intervention during which they receive training in two key metacognitive abilities: (i) knowledge of cognition (i.e., the ability to identify problem-solving solutions, know how to apply these solutions, and be able to determine which possible solution would be best to use to solve a given problem) and (ii) regulation of cognition (i.e., the ability to plan the use of problem-solving strategies, monitor the success of these strategies during their implementation, and accurately evaluate the success of these strategies after their implementation). These activities are complemented by additional exercises designed to identify real-world situations in which these skills could be applied (i.e., transfer of knowledge/skills) as well as learning evidence-based strategies designed to address intervening variables that may hinder transfer of such skills to real-world settings. For example, a participant with low self-efficacy with regard to their ability to apply skills learned in session to real-world settings may receive training in identifying and correcting cognitive distortions that may sustain such low self-efficacy. Specific intervening variables addressed in MCR include mood dysregulation, arousal dysregulation, and low self-efficacy/motivation. All skills taught in MCR are practiced in session using computerized activities [25] with the goal of having participants experience success in the use of these skills in a low-risk environment (i.e., the therapeutic setting) before being asked to try to use these skills to address real-world challenges that are likely to be more challenging.

In its modified form, MCR-Q is a 13-session intervention that includes one psychoeducation session followed by 12 MCR sessions. In the psychoeducation session, participants are provided with counseling on (i) preparing to quit and (ii) dealing with withdrawal. With regard to preparing to quit, participants review common smoking triggers and specific strategies to cope with or avoid these triggers. Additionally, participants complete the START exercise developed by the U.S. Department of Health and Human Services [26] to help people prepare to quit smoking. This exercise involves (i) *S*etting a quit date, (ii) *T*elling others about your plans to quit smoking, (iii) *A*nticipating challenges associated with quitting, (iv) *R*emoving cigarettes and tobacco products from one's environment, and (v) *T*alking to one's medical provider about quitting. For this exercise, participants set a quit date to occur the following week in between the first and second MCR-Q sessions. With regard to dealing with withdrawal, participants received instruction in two sets of strategies for dealing with cravings: HALT and the 4 D's. HALT provides participants with education on four emotional states that may make an individual more susceptible to craving (i.e., hunger, anger, loneliness, and feeling tired [26]). The 4 D's are a set of strategies that individuals can use to cope with cravings to smoke (i.e., deep breathing, drink water, do something else, and delay starting smoking for 15 minutes [27]).

Following the psychoeducation session, individuals began six weeks of twice-weekly MCR-Q sessions. These sessions followed the traditional format of an MCR session with two exceptions. First, participants completed a smaller subset of the MCR computerized activities [25]; activities were selected due to their utilization of working memory skills—a cognitive ability commonly linked to tobacco use among individuals with SMI [28, 29]. Second, exercises focused on applying these skills to specific aspects related to quitting smoking (e.g., coping with cravings).

#### 2.2.2. Pharmacotherapy

Participants were given the opportunity to receive one or more cessation medications: bupropion, varenicline, or nicotine patches, gum, or inhalers. Choice of medication was determined using a shared decision-making model in which the participant and the prescriber collectively identified the approach they agreed would be best.

### 2.3. Procedures

**Figure 2:**
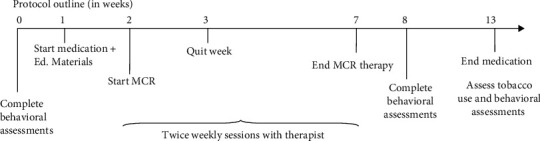
Study design.

Figure 2 displays the study timeline. Upon enrollment, participants completed a baseline assessment that confirmed their psychiatric diagnosis, measured tobacco use history and nicotine dependence, and established baseline values of psychiatric symptoms. Expired carbon monoxide (CO) was also measured. Next, participants met with a nurse practitioner to select a smoking cessation medication. After one week on this pharmacotherapy, individuals completed the MCR-Q psychoeducation session followed by twice-weekly MCR-Q sessions for 6 weeks. Following the completion of MCR-Q and six weeks later, participants' tobacco use, nicotine dependence, expired CO, and psychiatric symptoms were reassessed. Participants continued their pharmacotherapy regime throughout the MCR-Q sessions and for the six weeks that followed.

The proposed project was approved by the Ohio Department of Health Institutional Review Board, and all participated provided written informed consent to participate in the study.

### 2.4. Measures

#### 2.4.1. Assessments of Tobacco Use

Participants completed a self-report questionnaire with regard to their smoking behavior, including (i) years since becoming a regular smoker, (ii) number of cigarettes smoked each day, and (iii) number of 24-hour quit attempts. Expired CO, which provides an estimate of the level of CO in the blood stream, was assessed using a CO monitor. Finally, participants completed the Fagerström Test of Nicotine Dependence ((FTND): [30]) to assess the severity of nicotine dependence.

Occurrence of 24-hour quit attempts during the intervention period was tracked as an intermediate smoking outcome. Long-term outcomes included changes in expired CO, changes in FTND scores, reductions in smoking, and smoking abstinence. Consistent with current guidelines [31, 32], individuals were considered to be abstinent from smoking if they (i) reported not smoking any tobacco products during the past 7 days and (ii) provided an exhaled CO level < 5 ppm.

#### 2.4.2. Assessment of Participants' Perception of MCR-Q

At each MCR-Q session, participants completed the Session Rating Scale ((SRS): [33]) and the interest/enjoyment subscale of the Intrinsic Motivation Inventory for Schizophrenia Research ((IMI-SR): [34]). The SRS assesses four aspects of the therapeutic alliance as experienced during an individual psychotherapy session: (i) quality of the therapeutic relationship, (ii) agreement on goals and topics, (iii) perceived utility of the clinician's therapeutic approach, and (iv) overall quality of the completed session. Scores on the SRS range from 0 to 40, with scores ≤ 36 considered evidence of a possible suboptimal therapeutic alliance [35]. Modified from the original IMI [36, 37], the IMI-SR is a 21-item self-report scale designed to assess intrinsic motivation among individuals with schizophrenia [34]. While the entire scale is called the “Intrinsic Motivation Inventory,” the interest/enjoyment subscale is considered to be the only subscale that directly assesses intrinsic motivation [38, 39].

#### 2.4.3. MCR-Q Fidelity

Throughout the completion of the trial, 20% of the MCR-Q sessions were randomly selected for fidelity review. These sessions were audiotaped and reviewed by a member of the study team not involved in the delivery of MCR-Q or administration/scoring of study assessments using a modified version of the MCR Fidelity Scale [10]. Similar to the original MCR Fidelity Scale, the MCR-Q Fidelity Scale assesses the effectiveness of 10 aspects associated with the delivery of the MCR-Q—see Table 1. Each item is rated on a score from 1 to 5 with scores ≥ 4 considered to be indicative of high fidelity in the delivery of MCR-Q.

#### 2.4.4. Symptomatology Measures

The Positive and Negative Syndrome Scale (PANSS: [40]) was used to assess symptomatology among study participants. This clinician-rated measure assesses three domains of symptomatology: (i) positive symptoms, (ii) negative symptoms, and (iii) general symptomatology. Severity of depressive symptomatology was assessed using the Calgary Depression Scale for Schizophrenia (CDSS: [41]). All raters for the PANSS and CDSS completed a standardized training protocol prior to administering these instruments. As part of this training, raters coded 5 practice PANSS and CDSS interviews and achieved excellent levels of interrater reliability as compared to master ratings (all intraclass correlations ≥ 0.75).

### 2.5. Statistical Analyses

Total scores for the FTND were missing for 6 participants at baseline, 21 participants at 8-week follow-up, and 20 participants at 13-week follow-up. Likewise, exhaled CO values were missing for one participant at baseline and 18 participants at both 8-week and 13-week follow-up. For analyses of these data, we employed multiple imputation to address missing data.

Between-subject differences in categorical variables were evaluated using chi-square tests. Within-subject longitudinal changes in categorical variables were evaluated using McNemar's Test for 2-level categorical variables and Fleiss-Everitt chi-square test for ordered categorical variables with >2 levels. Consistent with statistical guidelines [42], in situations where the number of participants per cell in a 2 × 2 table is <5, mid-*p* values are reported. For analyses of multiply imputed categorical data, the median mid-*p* value across imputations is reported [43]. Within-subject, longitudinal changes in continuous variables were examined using Hedberg and Ayer's [44] regression-based test of within-subject change.

## 3. Results

Our sample was a near even distribution of men and women who were predominantly non-Latinx Black or non-Latinx White with a schizophrenia-spectrum disorder diagnosis (Table 2). Participants had smoked cigarettes for 31 years on average, and over 60% reported moderate or greater nicotine dependence. Utilization rates for different pharmacological options among participants are shown in Table 3, and engagement rates with MCR-Q and pharmacotherapy are shown in Table 4.

### 3.1. Smoking Outcomes

Nearly 80% of participants reported a 24-hour quit attempt during their participation in MCR-Q (Table 5)—a figure higher tha annual rates of serious quit attempts reported by individuals with psychotic-spectrum disorders in epidemiological studies (27-48%: [45–47]). It also exceeds the rate of serious quit attempts following participation in a web-based, motivational enhancing smoking cessation intervention for individuals with psychotics-spectrum disorders (30%: [48])—the only previous study to report rates of quit attempts among individuals with psychotic-spectrum disorders participating in a psychosocial smoking cessation intervention.

Individuals participating in the study reported a reduction FTND total scores at 8-week follow-up (*M* = 2.77) as compared to baseline (*M* = 5.14; *t* = −4.01; *p* < 0.01). Similarly, week 13 FTND total scores (*M* = 3.06) were also lower than baseline (*t* = −3.07; *p* = 0.03). Similarly, when FTND dependence scores were treated as an ordered categorical variable (i.e., low (total score = 0‐4), medium (total score = 5), and high dependence (total score = 6‐10)), participants were found to experience a reduction in their level of nicotine dependence from baseline to 8-week follow-up (*Fleiss-Everittχ*^2^ = 10.95; *p* < 0.01) and 13-week follow-up (*Fleiss-Everittχ*^2^ = 6.78; *p* = 0.01).

Individuals participating in the study evidenced a decline in exhaled CO from baseline (*M* = 27.04) to 8-week (*M* = 22.66; *t* = −2.45; *p* = 0.02) that was not mainteind at 13-week follow-up (*M* = 26.00; *t* = −0.38; *p* = 0.71).

At 8-week follow-up, there was a statistically significant increase from baseline in the number of individuals that were abstinent per expired CO (median mid-*p* = 0.03). However, this change was not maintained at 13-week follow-up (median mid-*p* = 0.13). For (i) self-reported abtinence and (ii) concurrent self-report and CO-verified abstinence, there were no statistically significant changes from baseline to 8-week or 13-week follow-up, respectively.

### 3.2. Participant Perception of MCR-Q

Across all sessions, the median SRS total score was always above the clinical cutoff. Additionally, for individual SRS items (possible range of scores: 0-10), the median rating for each item for each clinician was 10. In total, these data indicate that participants in MCR-Q established a strong and positive therapeutic alliance with the MCR-Q clinicians.

**Figure 3:**
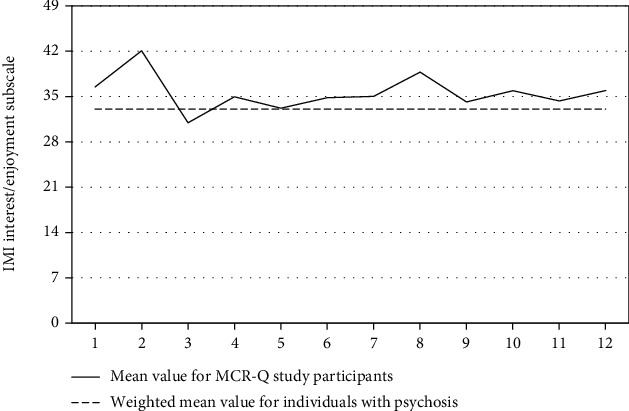
Intrinsic Motivation Inventory (IMI) scores across MCR-Q sessions.

Regarding the IMI, as there is no clinical cutoff for the Interest/Enjoyment subscale, we calculated a mean value for this subscale from all published trials reporting scores for this subscale among individuals with psychotic-spectrum disorders participating in psychosocial interventions [49–52]. To address variations in sample size across these studies, this mean value was weighted by the number of participants in each of these studies. Across all MCR-Q sessions, participants reported levels of intrinsic motivation similar to that reported among individuals with psychotic-spectrum disorders participating in other psychosocial interventions (Figure 3).

### 3.3. Treatment Fidelity

For each item and for the total score of the fidelity assessment, all ratings were ≥ 4, indicating that the intervention was delivered with high fidelity over the course of the study (Table 1).

### 3.4. Symptomatology

Following the completion of MCR-Q, participants reported a decline in positive symptoms as assessed by the PANSS (Table 6). There were no other statistically significant changes in PANSS scores or ratings of depression from the CDSS following the completion of MCR-Q.

## 4. Discussion

The results from the current study suggest cautious optimism with regard to the use of MCR-Q in combination with medication for individuals with psychotic-spectrum disorders who want to quit smoking. Despite the brevity of MCR-Q, at the completion of the intervention, participants experienced significant reductions in exhaled CO and self-reported severity of nicotine dependence on the FTND. Moreover, the number of individuals who made a 24-hour attempts to quit smoking during the intervention was dramatically higher than what would be expected based on epidemiological data, and nearly 25% of individuals reported a reduction in smoking at the end of MCR-Q. However, when abstinence was defined based on self-report or concurrent self-report and expired CO, there was no statistically meaningful change in smoking cessation at the completion of the MCR-Q intervention. Likewise, while improvements in nicotine dependence as assessed using the FTND were sustained when assessed five weeks after the completion of MCR-Q, reductions in expired CO were not. These conflicting data raise questions about the ability of the intervention as currently designed to produce meaningful and durable reductions in tobacco use among individuals with psychotic-spectrum disorders. It may be that an expanded version of MCR-Q that provides smoking cessation support for a longer duration may be required to produce greater rates of smoking cessation that are sustained after the completion of the intervention. Such an intervention would be consistent with the original design of MCR in which the intervention is delivered for a considerably longer duration than MCR-Q (i.e., six months versus 8 weeks).

Individuals with psychotic-spectrum disorders report several negative experiences with regard to existing smoking cessation interventions, including perceived limited efficacy of both pharmacological and psychosocial treatments [53]. In light of these findings, our data with regard to ratings of therapeutic alliance and intrinsic motivation during MCR-Q are especially encouraging. With regard to therapeutic alliance, SRS scores at all MCR-Q sessions were indicative of a positive therapeutic relationship in which participants viewed the interventions as aligning well with their goal to quit smoking and providing the supports that they needed to achieve this goal. Moreover, despite the inherent challenges in quitting smoking, data from the IMI suggest that participants' intrinsic motivation to participate in MCR-Q is comparable to levels of intrinsic motivation for participation in other psychosocial interventions among individuals with psychotic-spectrum disorders.

Of note, the study does suffer from several limitations. For example, the lack of a control group and the short follow-up interval in our study prevents definitive conclusions with regard to the benefits of the intervention. Likewise, participants in our study had been smoking regularly for, on average, over 30 years. Given this extensive smoking history, we may have been limited in our ability to facilitate smoking cessation in a population in which this behavior was so engrained. Investigation of MCR-Q among individuals with psychotic-spectrum disorders with a shorter smoking history may clarify whether the benefits of this intervention are greater when provided sooner following the start of regular smoking. Finally, participants in our study reported high rates of cigar and marijuana smoking (in addition to cigarettes) that may have contributed to higher CO values at follow-up despite reported reductions in cigarette use.

Despite growing advances in the treatment of psychotic-spectrum disorders, the mortality gap between individuals with psychotic-spectrum disorders and those without appears to be increasing over time [54]. Increased rates of smoking among individuals with psychotic-spectrum disorders have been identified as a key contributor to this lifespan reduction [55]. Consequently, continued development and dissemination of smoking cessation interventions to individuals with psychotic-spectrum disorders may be a key strategy to improve both the quantity and quality of years lived by individuals with these disorders.

## Figures and Tables

**Table 1:** MCR-Q fidelity ratings.

Measure	Avg rating
Interpersonal effectiveness and therapeutic alliance	4.5
Promotion of knowledge about cognition	4.3
Promotion of regulation of cognition	4.1
Intervening factors	4
“Real world” application	4.1
Task order and passing criteria	5
Introduction to a new module	5
Introduction and feedback	4
Time management	4.9
Session frequency and duration	4.4
Average score across items	4.4

**Table 2:** Participant demographics.

Variable	Mean ± SD or %
Male gender	54.2%
Age	50.0 ± 7.9
Race/ethnicity	
Non-Latinx White	42.6%
Non-Latinx Black	44.7%
Other	12.7%
Diagnosis	
Bipolar disorder with psychotic features	29.3%
Schizophrenia/schizoaffective	70.7%
Years since becoming regular smoker	31.0 ± 11.7
Time to first cigarette	
Within 5 minutes	66.7%
6-30 minutes	22.9%
31 or more minutes	10.4%
Smoking frequency	
10 or fewer cigarettes per day	36.2%
11-20 cigarettes per day	40.4%
21 or more cigarettes per day	21.3%
Fagerström Test of Nicotine Dependence	
Low dependence (0-4)	37.2%
Moderate dependence (5)	19.6%
High dependence (6-10)	44.2%
Number of quit attempts in past year	1.1 ± 1.7

**Table 3:** Utilization of pharmacological treatments for smoking cessation among MCR-Q participants.

Pharmacological treatment	*n* (%)
Multiple treatments	16 (33%)
Bupropion	14 (29%)
Nicotine replacement therapy	10 (20%)
None	7 (14%)
Varenicline	2 (4%)

**Table 4:** Engagement with MCR-Q and pharmacotherapy and completion of follow-up assessments.

Measure	*n* (%)
Engagement with intervention	
MCR-Q	
100% of sessions completed	32 (66.7%)
81-99% of sessions completed	2 (4.1%)
50-80% of sessions completed	3 (6.3%)
< 50% of sessions completed	11 (22.9%)
Pharmacotherapy	
81-100% of sessions where pharmacotherapy was used	33 (68.8%)
50-80% of sessions where pharmacotherapy was used	6 (12.5)
< 50% of sessions where pharmacotherapy was used	3 (6.3%)
Pharmacotherapy was never used	6 (12.5%)

Follow-up	
8-week follow-up	
Completed	33 (68.7%)
No show	12 (25.0%)
Withdrew from study	3 (6.3%)
13-week follow-up	
Completed	31 (64.6%)
No show	14 (29.1%)
Withdrew from study	3 (6.3%)

**Table 5:** Smoking-related outcomes.

Measure	*n* (%)
At least one quit attempt during study period	38 (79.2%)
*Confirmed abstinence (per CO levels and self-report)*	
8 weeks	3 (6.3%)
13 weeks	2 (4.2%)
*Smoking reduction*	
8 weeks	11 (22.9%)
13 weeks	9 (18.8%)
*CO levels*	
Baseline	26.90
8 weeks	22.83
13 weeks	25.59

**Table 6:** Symptomatology ratings over course of treatment.

	Baseline (mean ± SD)	Week 8 (mean ± SD)	*p* value
Positive and Negative Syndrome Scale			
Positive	14.8 ± 5.7	12.1 ± 3.9	0.02
Negative	14.0 ± 5.0	13.3 ± 5.7	0.99
General	28.0 ± 7.4	26.8 ± 7.5	0.88
Calgary Depression Scale	5.0 ± 4.1	3.0 ± 4.2	0.22

## Data Availability

The datasets generated and/or analyzed during the current study are not publicly available due to participants not providing consent to do so but are available from the corresponding author on reasonable request.
